# Improvement of Paraneoplastic Limbic Encephalitis after Systemic Treatment with Rituximab in a Patient with B-Cell Chronic Lymphocytic Leukemia

**DOI:** 10.1155/2013/958704

**Published:** 2013-08-01

**Authors:** Hendrik Nogai, Heike Israel-Willner, Rolf Zschenderlein, Antonio Pezzutto

**Affiliations:** ^1^Department of Hematology, Oncology, and Tumor Immunology, Charité-Universitätsmedizin Berlin, Campus Benjamin Franklin, Hindenburgdamm 30, 12203 Berlin, Germany; ^2^Department of Neurology, Charité-Universitätsmedizin Berlin, Campus Charité Mitte, Charitéplatz 1, 10117 Berlin, Germany

## Abstract

Limbic encephalitis is an inflammatory disease of the central nervous system characterized by diverse neurologic symptoms including mnestic disturbances, hallucinations, and seizures as well as behavioral symptoms like depression, personality changes, and acute confusional states resembling dementia. Several antibodies have been described in the pathogenesis of limbic encephalitis. It is often a paraneoplastic syndrome associated with small cell lung cancer, breast cancer, or Hodgkin's lymphoma among others. Here, we report a patient with B-cell chronic lymphocytic leukemia (B-CLL), presenting with otherwise unexplained neurologic symptoms consistent with limbic encephalitis. Despite intensive diagnostic procedures, no causing agent could be identified. Pleocytosis consisting of T cells was detected in the cerebrospinal fluid (CSF). We initiated anti-B-cell therapy with Rituximab for B-CLL with quick and durable resolution of symptoms. We speculate that disruption of interaction between autoreactive T and malignant B cells is responsible for the therapeutic effect of Rituximab.

## 1. Case Presentation

A 77-year-old patient with B-cell chronic lymphocytic leukemia was admitted to our hospital for evaluation of a progressive deterioration of his general condition, ataxia, and signs of a delirious syndrome in October 2007. The patient's wife had noticed personality changes and intermittent disorientation for approximately three weeks prior to admission.

The CLL had been diagnosed in 2001 and had shown an indolent course. The leukemic cells were ZAP70 and CD38 negative. A biweekly low-dose therapy with chlorambucil had been started in June 2007 when the leucocyte count had reached 200.000/*µ*L. Hemoglobin value was 11.2 g/dL; the platelet count was 226.000/*µ*L. With this therapy, the leucocytes had slowly decreased to 106.000/*µ*L in September 2007, when the treatment was stopped. At the time of admission, there was no palpable lymphadenopathy or systemic symptoms. The spleen was slightly enlarged; a moderate hypogammaglobulinemia was present. The further medical history included chronic obstructive pulmonary disease and lumbar spinal stenosis. Seven years before the present admission, the patient had suffered a transient cerebral ischemia without any residual neurological deficiency.

**Figure 1:**
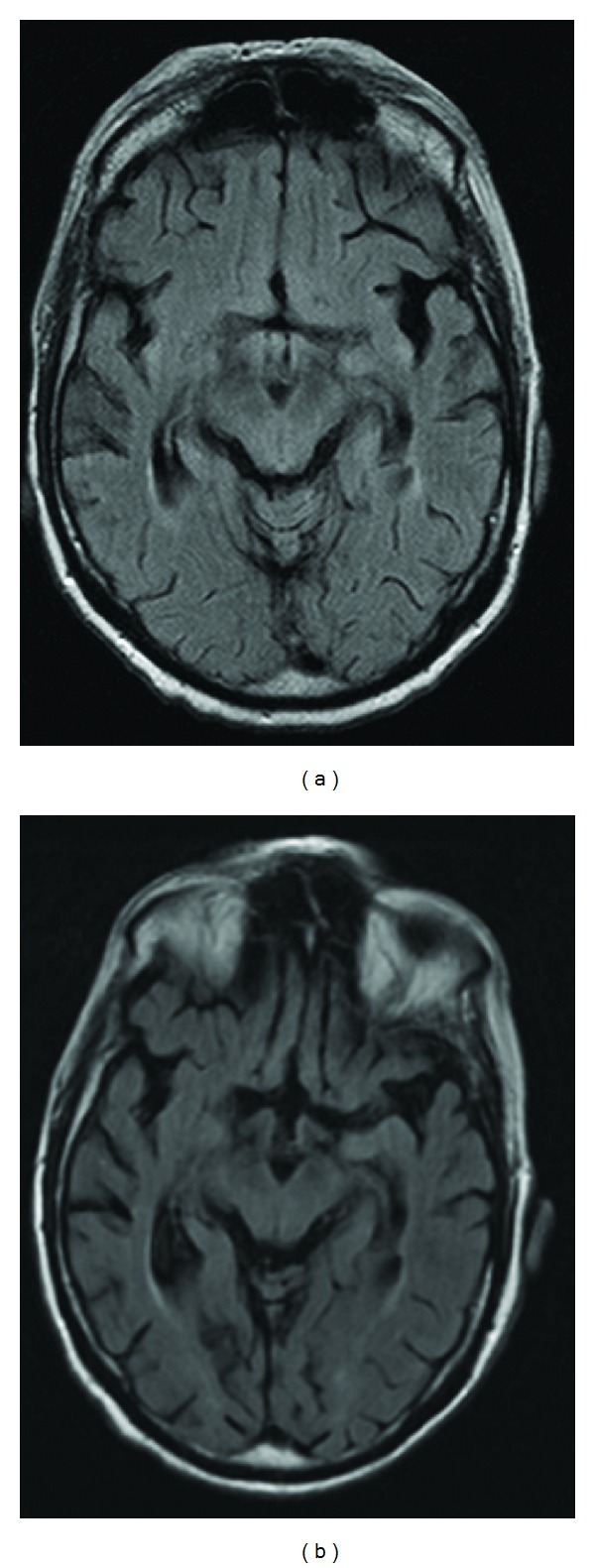
Axial FLAIR images obtained before initiation of Rituximab. Normal anatomy and signal intensity of limbic system were seen at initial presentation as well as after longer course of disease (left: November 2007; right: February 2008).

At initial evaluation, the patient showed mnestic disturbances and fluctuating disorientation with aphasic phases, as well as severe ataxia. No focal abnormalities were found at neurological examination. Cerebral computed tomography (CT) scan and contrast magnetic resonance imaging (MRI) showed no signs of acute cerebral ischemia, encephalitis, or meningeal enhancement (Figure 1). The electroencephalogram (EEG) was consistent with a general cerebral dysfunction, but without focal changes. Examination of the CSF demonstrated a massive lymphocytic pleocytosis (768 cells/*µ*L) composed almost exclusively of CD3+ cells, and elevated protein (130 mg/dL), with no evidence of oligoclonal bands but rather of intrathecal IgM synthesis. Unfortunately, flow cytometric evaluation of CSF to further differentiate T-cells was not performed. No infectious agents, especially no herpes simplex virus, varicella zoster virus, Epstein-Barr virus, cytomegalovirus, parvovirus, enterovirus, adenovirus, toxoplasma, mycoplasma, borrelia, or lues, could be detected. There were no atypical cells in repeated CSF analyses. The serum tested negative for paraneoplastic antibodies against Hu, Yo, Ri, Amphiphysin, CRMP5, Ma-1, Ma-2, CV2, and voltage-gated potassium channels. A CT scan of the chest and abdomen revealed ubiquitous slightly enlarged lymph nodes, attributed to the CLL, but no signs of other malignancies. 

Rehydration and empirical antibiotic therapy with cefuroxime for a documented urinary tract infection showed transient and modest improvement of the intermittent confusion, but cognitive symptoms persisted. Neuropsychological examination revealed a dysexecutive syndrome and an impaired short-term memory. 

At this point, no definite diagnosis could be established. Viral encephalitis (with unknown pathogen) was suspected and no specific treatment was initiated. The patient was admitted to a rehabilitation clinic for physiotherapeutic treatment.

Within the following weeks, both the physical and the mental condition of the patient further worsened, and he developed a wasting-like syndrome with a weight loss of approximately 20 kg within two months, with severe inappetence, gait disturbances, and persisting cognitive dysfunction resembling dementia.

Because of the progressive clinical symptoms in the absence of any evidence for other diseases than the CLL and the findings in the cerebrospinal fluid, a tentative diagnosis of limbic encephalitis, possibly paraneoplastic, was established.

Due to the severely reduced performance status of the patient rendering any intensive therapy impossible and considering its activity against both B-cell malignancies and some autoimmune conditions, we initiated a weekly systemic treatment with the monoclonal CD20 antibody, Rituximab. After four times of systemic administration of Rituximab (375 mg/m²), we noticed a substantial improvement of the neurologic symptoms and a decline in cells in the CSF, still composed of CD3+ T cells (from initial 768/*µ*L to 300/*µ*L). The peripheral lymphocyte count dropped to normal values. The total WBC was 3.500/*µ*L with >70% neutrophils; hemoglobin and platelets were within normal limits.

Since then and without any further specific treatment, the condition of the patient completely recovered. After more than three years, there was no evidence of neurologic impairment and the CLL was not detectable in peripheral blood. The patient was in a very good condition and underwent successful surgery for spinal stenosis. However, he deceased due to postoperative complications unrelated to CLL or the limbic encephalitis following surgical prostatectomy in a community hospital.

## 2. Discussion

Cerebral or meningeal involvement is a very rare event in patients with CLL. The most common clinical presentations are confusional state, meningitis with cranial nerve abnormalities, optic neuropathy, and cerebellar signs [1]. Neither of those was present in our patient. 

Another rare brain dysfunction in CLL patients is the occurrence of progressive multifocal leukoencephalitis (PML) [2]. The frequency of this disease, which is due to infection with the polyoma virus JC, is increasing because of the wider use of effective immunosuppressants and particularly purine analogues. In our patient there was neither evidence of leukemic cells in the CSF nor evidence of JC virus infection. The MRI also lacked focal cerebral lesions typical in PML. 

Limbic encephalitis is characterized by rapid development of irritability, depression, sleep disturbances, seizures, hallucinations, and short-term memory loss. The clinical manifestations of limbic encephalitis are diverse and patients often present with a puzzling clinical picture. Delayed diagnosis is common.

Limbic encephalitis was first considered to be a disorder that almost always occurs in association with malignancies, but several cases unrelated to cancer have been described. Its occurrence has been associated with antibodies against intracellular neuronal antigens (e.g., Anti-Hu, Anti-Ma2, Anti-Yo, and CV2/CRMP5) or against cell membrane antigens (e.g., voltage-gated potassium channel, VGKC; N-methyl-D-aspartate receptors, NMDAR) [3]. In approximately 40% of patients, no antibodies can be detected, so their absence does not preclude the diagnosis [4]. Importantly, this finding raises the possibility that the disease might be mediated by cellular immunity. A mixed cellular and humoral pathogenetic mechanism is not uncommon in autoimmune diseases.

Radiologic criteria of paraneoplastic limbic encephalitis include MRI FLAIR or T2 uni- or bilateral temporal lobe hyperintensities [4]. Imaging criteria are facultative and it is not unusual that patients with a syndrome typical of limbic encephalitis have normal MRI studies. Conversely, it is possible that the clinical picture is atypical, but the MRI shows bilateral medial temporal lobe abnormalities [5]. In our case, MRI showed no specific signs of limbic encephalitis. 

Paraneoplastic limbic encephalitis (PLE) is most frequently associated with small cell lung cancer but has also been described in patients with other solid tumors. PLE linked to Hodgkin's lymphoma with antibodies against metabotropic glutamate receptor 5 (mGlu-R5) is termed Ophelia Syndrome. Association with non-Hodgkin's lymphoma is rare [6–8]. To our knowledge, no case of chronic lymphocytic leukemia with PLE has been described so far. One case of T-cell pleocytosis in the CSF in a patient with B-CLL who suffered from progressive somnolence and blurred vision has been reported [9]. According to the case report, no MRI was performed neither had the autoantibodies been tested.

Our patient improved markedly after the initiation of treatment with Rituximab. In the light of the severe, quickly progressive deterioration of the patient's condition until initiation of anti B cell-therapy, we strongly assume a therapeutic effect of Rituximab rather than spontaneous improvement. The mechanism of action remains debatable. 

The use of Rituximab and the following CD20 depletion may have altered the interaction between B and T cells. Rituximab-induced elimination of circulating B cell results in significant clinical improvement in rheumatoid arthritis [10]. In this disorder, B cells may function as antigen-presenting cells and are important for T-cell activation. Although such a role for B cells has been demonstrated neither in CLL nor in paraneoplastic syndromes, it has been known for long time that quantitative and qualitative abnormalities of T cells are present in B-CLL [11]. Recently, a murine model suggesting a crucial role of T cells in CLL has been reported [12].

It is possible that improvement of the encephalitis in our patient is due to a disruption of the interplay between neoplastic B cells and reactive T cells. Such a mechanism has been assumed in some variants of PLE associated with autoantibodies such as anti-VGKC or anti-NMDAR, where T cells are considered to play a role in antibody induction.

In an uncontrolled trial, a total of nine patients with paraneoplastic syndromes, including one patient with PLE, were treated with Rituximab [13]. Three patients showed a response to the treatment with improvements of neurological syndromes. Two of them had a small cell lung cancer that responded completely to the concurrent chemotherapy.

Although we cannot formally exclude the fact that our patient suffered from unidentified viral encephalitis, the clinical course with the rapid improvement of the neurological symptoms in conjunction with the near complete remission of the CLL is highly suggestive of paraneoplastic CLL-related limbic encephalitis. In our opinion, CLL must be included among the malignancies possibly related to limbic encephalitis. Considering the older age of many CLL patients this condition might be underdiagnosed, being possibly confused with other common causes of brain dysfunction in older patients, such as Alzheimer's disease or dementia.
